# 5-year clinical outcome of the ESTOIH trial comparing the short-bite versus large-bite technique for elective midline abdominal closure

**DOI:** 10.1007/s10029-025-03459-9

**Published:** 2025-08-29

**Authors:** René H. Fortelny, Petra Baumann, Anna Hofmann, Stefan Riedl, Jan Ludolf Kewer, Jessica Hoelderle, Andreas Shamiyeh, Bettina Klugsberger, Theo David Maier, Guido Schumacher, Ferdinand Köckerling, Guido Wöste, Ursula Pession, Markus Albertsmeier

**Affiliations:** 1 Wilhelminenspital, Allgemein-, Viszeral- Und Tumorchirurgie, Montleartstr. 37, 1160 Vienna, Austria; 2 Department of Medical Scientific Affairs, Aesculap AG, Am Aesculap Platz, 78532 Tuttlingen, Germany; 3 Alb Fils Klinik GmbH, Klinik Am Eichert, Allgemeinchirurgie, Eichertstr.3, 73035 Göppingen, Germany; 4 Klinikum Landkreis Tuttlingen, Klinik Für Allgemein-, Viszeral- Und Gefäßchirurgie, Zeppelinstr. 21, 78532 Tuttlingen, Germany; 5 Kepler Universitätsklinikum GmbH, Klinik Für Allgemein- Und Viszeralchirurgie, Krankenhausstr. 9, 4021 Linz, Austria; 6 Robert-Bosch-Krankenhaus, Allgemein- Und Viszeralchirurgie, Auerbachstr. 110, 70376 Stuttgart, Germany; 7 Städtisches Klinikum Braunschweig, Chirurgische Klinik, Salzdahlumer Str. 90, 38126 Brunswick, Germany; 8 Vivantes Klinikum Spandau, Klinik Für Chirurgie, Viszeral-, Und Gefäßchirurgie, Neue Bergstr. 6, 13585 Berlin, Germany; 9 Universitätsklinikum Frankfurt, Zentrum Der Chirurgie, Klinik Für Allgemein- Und Viszeralchirurgie, Theodor-Stern-Kai, 60590 Frankfurt Am Main, Germany; 10 Department of General, Visceral and Transplantation Surgery, Ludwig-Maximilians-Universität (LMU) Munich, LMU University Hospital, 81377 Munich, Germany; 11 Sigmund Freud Privatuniversität, Med. Fakultät, Freudplatz 3, 1020 Vienna, Austria

**Keywords:** Randomised controlled trial, Incisional hernia, Suture stitch technique, Suture bite technique, Abdominal wall closure, Laparotomy

## Abstract

**Background:**

The short-bite technique for fascial closure after midline laparotomy has been shown to reduce the incidence of incisional hernias one year postoperatively compared to the traditional large-bite technique. However, most studies evaluating this approach have been limited to a one-year follow-up period. Initiated in 2013, the ESTOIH trial is the only randomised controlled study to include both 3-year and 5-year follow-up data. The 3-year clinical outcomes have been previously published. Herein, we report for the first time the 5-year results regarding the incisional hernia rate using the small-bite technique compared to the large-bite technique for elective midline closure.

**Methods:**

The ESTOIH study was designed as a prospective, multicentre, parallel, double-blind, randomised controlled study of primary elective midline closure. Patients were randomly assigned to receive either the small-bite or large-bite technique to close the fascia using an ultra-long-term, absorbable, elastic, monofilament suture named Monomax® based on poly-4-hydroxybutyrate. A planned 5-year follow-up was conducted, including ultrasound/radiological imaging to assess incisional hernia development as a key outcome parameter for the long-term effectiveness of the procedure.

**Results:**

In total, 362 patients were included in the 5-year ITT analysis (175 and 187 patients in the short-bite and large-bite groups, respectively). The incisional hernia rate increased in the short-bite group from 7.58% to 9.14% (p = 0.58) and in the large-bite group from 10.45% to 13.90% (p = 0.30) after 5 years compared to 3 years postoperatively. The incisional hernia rate in the short-stitch group was low, at 9.14% five years after surgery; however, the difference between the two treatment groups (short vs. long) was not significant at 5 years (OR 1.60, 95% CI [0.82–3.10]; p = 0.155).

**Conclusion:**

The previously observed increase in incisional hernias from 1 to 3 years postoperatively continued to 5 years in both stitch groups. The incisional hernia rate in the long-stitch group appeared to be higher at every time point than that in the short-stitch group. Using the short-bite technique in combination with an extra-long-term absorbable, elastic, monofilament poly-4-hydroxybutyrate suture, it may be possible to achieve a very low incisional hernia rate in the long-term follow-up.

**Trial registry:**

NCT01965249, registered October 18, 2013.

## Introduction

One-third of the population in industrialised countries will undergo abdominal surgery in their lifetime [1]. Burst abdomen is the most feared short-term complication, and incisional hernia is the most commonly observed long-term complication after midline incision. The incidence of incisional hernia development is reported to be 30% two years and up to 60% five years after surgery, depending on the patient's risk factors, the type of incision used to open the abdominal cavity, surgical settings (elective or emergency), and the choice of suture technique for abdominal wall closure [2]. This implies that the incidence of incisional hernia has been significantly underestimated in previous trials with a short follow-up of one year; therefore, an extended follow-up is essential to draw conclusions about the best surgical approach [3].

The Inline Meta-Analysis published in 2010 indicated that a long-term absorbable monofilament in the continuous suture technique should be favoured to close elective median laparotomies [4]. A Cochrane Review published in 2017 confirmed this finding; however, no recommendation was made regarding the short bite technique [5]. The first meta-analysis that compared short versus long stitches for abdominal wall closure was the MATCH Review [6]. A subgroup analysis of two randomised controlled trials showed that the incisional hernia rate was significantly lower in the short-bite group than in the long-bite group [7, 8]. The authors concluded that a long-term, absorbable monofilament suture should be applied using the continuous short-bite technique to decrease the incidence of incisional hernias.

The recent European Hernia Society (EHS) guidelines for elective median abdominal wall closure currently contain a strong recommendation regarding the use of a continuous suture. In contrast, only a weak recommendation can be made regarding the short-bite technique and suture material due to the lack of available evidence [9].

Previous studies have used a polydioxanone-based suture as a long-term absorbable monofilament suture material. This type of suture material is absorbed in the human body within 180 days postoperatively, and it also loses 50% of its tensile strength during the first 30 days [10]. However, tt is known that the abdominal fascia regains only 70% of its initial strength 1 year after primary laparotomy [11]. Data have also shown that 52% of incisional hernias occur within 6 months after surgery, 60% within 1 year, and 79% within two years postoperatively [11, 12], which implies that the absorption rate of traditional suture materials might be too fast, and prolonged suture support may be needed for adequate abdominal wall closure [11]. Therefore, a new ultra-long-term absorbable, elastic, monofilament suture material named"Monomax"made of poly-4-hydroxybutyrate homopolymer was developed in 2007 and analysed for the first time in human beings regarding its safety and efficacy in the ISSAAC trial [13, 14]. This suture material combines high tensile strength with a high degree of pliability among the currently available absorbable monofilament sutures. It exhibits several properties relevant to clinical use, including high elasticity, prolonged strength retention, low tissue drag, and reliable knot security. These characteristics align with key criteria for an effective suture material for midline abdominal wall closure. Animal tests demonstrated that this type of suture material exhibited excellent biocompatibility and good physiological tissue integration, without causing tissue irritation or infection [13]. These properties have been confirmed in the ISSAAC multicentric, prospective, human clinical trial, and the results also indicated that the suture material is safe and efficient for abdominal wall closure after primary midline laparotomy [15].

A second multicentric, international, prospective trial (MULTIMAC) performed under clinical routine settings proved the safety and effectiveness of the poly-4-hydroxybutyrate suture material for midline as well as for transverse abdominal wall closures even in high-risk patients (obese, AAA, and diabetes patients) regarding the short-term complications such as burst abdomen and wound infections [16]. Since both the ISSAAC and MULTIMAC studies utilized the poly-4-hydroxybutyrate suture material in the long-stitch technique for fascia closure, a randomised controlled trial named ESTOIH was designed in 2013 to compare the short-bite technique with the long-bite technique in combination with the poly-4-hydroxybutyrate suture material regarding short- and long-term clinical outcomes [17]. Based on the need for a significantly longer follow-up period, the ESTOIH study is the first RCT to analyse small bites versus large bites for fascia closure, including a five-year postoperative examination. Short-term results, focusing on burst abdomen and surgical site infections, showed a trend toward a lower burst abdomen rate in the short-stitch group, implying that the elastic, ultra-long-term absorbable monofilament was associated with an overall low complication rate, including wound infections [18]. Long-term follow-up findings of the ESTOIH study 1 and 3 years after surgery showed that at both time points, the incisional hernia rate was lower and the quality of life significantly higher in the small-bite group than in the large-bite group [19, 20].

In this article, we report for the first time the clinical findings obtained after five years regarding the comparison of small bites versus large bites for elective midline laparotomy closure using an elastic, ultra-long-term absorbable, poly-4-hydroxybuyrate, monofilament suture material.

## Methods

We have published our a priori study protocol of the ESTOIH study, in which the study design, participant inclusion and exclusion criteria, intervention details, randomisation method, and statistics were described [17]. Furthermore, the ESTOIH clinical short-term results obtained 30 days postoperatively, as well as the 1-year and 3-year outcomes, have been reported previously [18–20].

### Trial design

For the ESTOIH trial, a multicentre, double-blind, controlled design was chosen using 1:1 randomisation. The participating centres were located in Germany and Austria.

This study was registered at ClinicalTrials.gov on October 13 2013 (NCT01965249). Before the start of the trial, ethics approval was obtained from all institutional review boards responsible for the participating clinics. This study was conducted in accordance with the ethical standards outlined in the 1964 Declaration of Helsinki and its subsequent amendments.

### Participants

The inclusion criteria for the trial were as follows: age ≥ 18 years, American Society of Anesthesiologists score I -III, patients who underwent a primary midline laparotomy with an incision length of ≥ 15 cm for abdominal surgery, and an expected survival of at least one year after surgery.

The exclusion criteria were as follows:

- Emergency surgery.

- BMI ≥ 30 kg/m.^2^

- Surgery for abdominal aortic aneurysm and pancreatic tumours,

- Patients with the following conditions: peritonitis, coagulopathy, immunosuppressive therapy at the time of surgery (more than 40 mg of a corticosteroid per day or azathioprine), chemotherapy within the last two weeks before surgery, or abdominal radiation therapy within the last eight weeks before surgery.

- Pregnant women.

- Patients with severe neurologic and psychiatric diseases or poor compliance.

All participants provided written informed consent before inclusion in the study. The demographic and clinical characteristics of the patients, including baseline comorbidities, were recorded before surgery.

Recruitment was lower than expected; therefore, two changes were made to the original study protocol: The BMI exclusion criterion of ≥ 30 kg/m^2^ (meant to ensure homogeneity of the study cohort) was dropped, and the exclusion criterion"pancreatic tumour patients"(meant to exclude patients who would most likely not complete follow-up) was changed to"pancreatic cancer patients"to allow the inclusion of patients with benign tumours.

Patient recruitment was conducted at seven German and two Austrian hospitals. Of these centres, three were university hospitals, three were tertiary referral centres, and three were local and regional hospitals.

### Interventions

The skin and subcutis were incised, followed by the removal of the subcutaneous adipose tissue from the linea alba to an extent of at least 1 cm in all directions. It was a standard practice in the ESTOIH study to dissect the umbilical stalk from the aponeurosis, and refixation was performed after the fascia was closed. In both stitch technique groups, the same suture material consisting of poly-4-hydroxybutyrate (Monomax®) manufactured by B.Braun Surgical S.A.U., Barcelona, Spain, was used to close the rectus fascia. The physical properties of the Monomax® suture material and a comparison with other suture materials used for abdominal wall closure have been published previously [13].

The large-bite group was characterised by stitch intervals of 10 mm and a 10 mm distance from the wound edge using Monomax® USP 1, 150 cm loop with an HR 48 mm needle, which should lead to a suture length-to-wound length ratio (SL:WL ratio) of 4:1. For the small-bite technique, a single, continuous suture was used for fascia closure with 5 mm intervals between stitches and a 5–8 mm distance from the wound edge using a Monomax® USP 2/0 150 cm thread combined with a HR 26-mm needle. The resulting SL:WL ratio should be at least 5:1 or greater.

Before recruitment started, surgeons were trained on-site by the coordinating investigator (RHF) and instructed to use training videos. The number of throws per knot was not specified, but a minimum of six throws for the long-stitch technique and the use of a self-fixing knot at the start and end of each suture row in the short-stitch technique were recommended during training sessions. The number of stitches was counted, and the time required to close the fascia was measured using a stopwatch by either a study nurse or another assistant intraoperatively. For each study participant, the intraoperative parameter characterising the suture technique (SL:WL ratio) was documented in a case report form after surgery, and the following details were collected: length of the incision, number of threads, number of stitches, length of the implanted thread, and length of the remaining thread. These were reviewed during regular visits by a monitor at each study site to ensure homogeneity of the suture technique in all participating centres.

### Outcome measures

#### Primary outcome

The incisional hernia rate one year after surgery was the primary endpoint of the study, using the definition of the European Hernia Society (EHS)—"abdominal wall hernia with or without protrusion in the area of the postoperative scar that is perceptible or palpable by clinical examination or imaging"[3]. Further follow-up visits were performed after 3 and 5 years. For incisional hernia diagnosis, the patients were physically examined, followed by ultrasound imaging of the abdominal wall. In cases where routine cross-sectional imaging (CT or MRI) was indicated during the follow-up visit, no additional ultrasound examination was performed.

#### Secondary outcomes

As secondary safety parameters, short-term complications such as surgical site infections (SSI), ruptured abdominal wounds, wound healing disorders, seromas, haematomas, and other adverse events not directly related to wound healing were analysed until 30 days after surgery and have been published previously [17]. As a further effectiveness variable, the quality of life was assessed by using the EQ-5D-5L questionnaire [4] preoperatively and at 30 days, 1 year, 3 years, and 5 years postoperatively.

### Sample size calculation

For the sample size calculation, the results of the ISSAAC study [15] were used, which reported a 19% risk of incisional hernia development after one year for the long-bite technique. The aim of the ESTOIH study was to demonstrate that the application of the short-bite technique could reduce the incisional hernia rate by 50% at 1 year after surgery compared to the long-bite technique. A sample size calculation indicated the inclusion of 424 patients (212 per group) when the power was set to 80% with an alpha error of 5% and an assumption of an absolute risk reduction from 19% to 9.5%. Considering a dropout rate of 10%, the total number of patients to be randomised was 468. Patients who dropped out were not replaced in the study. To avoid centre-specific effects, the total number of patients who could be recruited at a centre was limited to 200. Recruitment was stopped after the randomisation of 424 patients based on an interim analysis of the primary outcome parameter.

### Randomisation

Randomisation was performed intraoperatively just before fascia closure using sealed opaque envelopes containing information about the suture technique and required suture material depending on the randomisation result. The sponsor prepared the randomisation envelopes according to a randomisation list created by a statistician using SAS 9.1 statistical software (SAS Institute Inc., Cary, NC, USA). Patients were randomly allocated in a 1:1 ratio to the small-bite or large-bite group. To prevent centre-specific effects and guarantee a balanced treatment distribution within the centres, a separate randomisation list was generated for each clinic. Different random blocks of varying lengths were chosen by the statistician, and the randomisation lists were sealed and stored at the sponsor site.

### Blinding

The ESTOIH study used a double-blinded technique, meaning that the patients and observers evaluating the postoperative clinical outcomes were unaware of the suture technique allocation. The assessors were unable to access the randomisation list, and for the evaluation of the clinical outcome, a study nurse or an independent person handed over the case report form for documentation by the assessor. Surgeons performing the abdominal wall closure could not be blinded, but they were not involved in outcome evaluation.

## Results

### Patients

Recruitment took place between March 2014 and December 2019. In total, 425 patients were randomised and allocated to the small-bite group (n = 215) or the large-bite group (n = 210). No differences were observed in demographic and baseline data, including comorbidities such as BMI, diabetes, COPD, ASA classification, and smoking status, in either treatment group of the ESTOIH population. These data have been published previously [18]. Furthermore, the short-term outcomes of the ESTOIH study, including the burst abdomen and surgical site infection rates until 30 days postoperatively, have already been reported [18]. In addition, findings regarding the incisional hernia rate 1 and 3 years after surgery have been published in previous articles [19, 20].

**Fig. 1:**
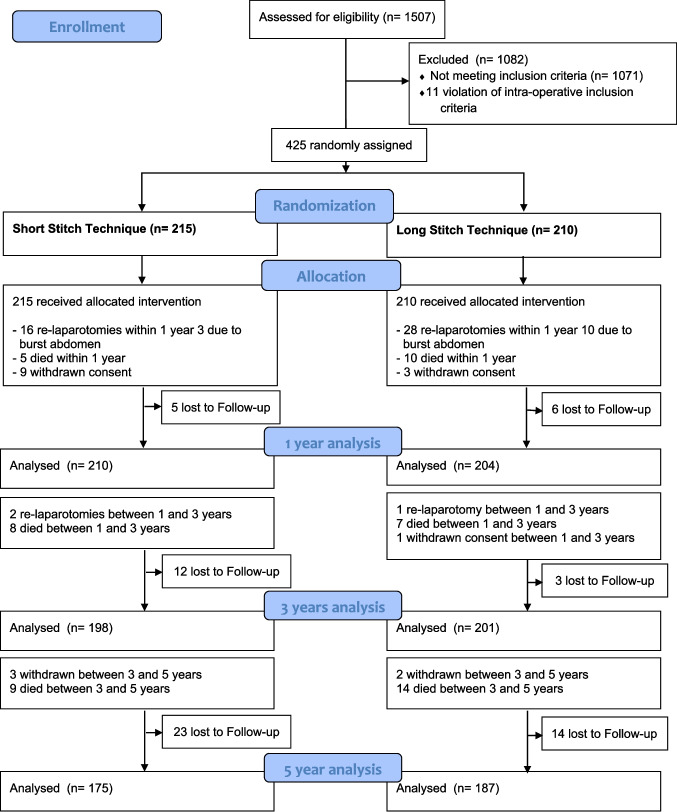
CONSORT Flow Diagram ESTOIH 5 years

The complete CONSORT flowchart for up to 5 years postoperatively is shown in Fig. 1.

Table 1 shows the incisional hernia rate of the ESTOIH intention-to-treat (ITT) and per-protocol (PP) populations analysed after 1, 3, and 5 years. At 1 year after surgery, the ITT analysis included 414 patients (short-bite group, n = 210; large-bite group, n = 204), and the PP analysis included 323 patients (short-bite group, n = 165; large-bite group, n = 158). A decrease was observed in the number of patients analysed in the ITT population (n = 399; 198 and 201 patients in the small-and large-bite groups, respectively) and in the PP population (n = 273; small-bite group, n = 139; large-bite group, n = 134) 3 years postoperatively. Five years after surgery, 362 patients were available for the ITT analysis (small-bite group, n = 175; large-bite group, n = 187), and 216 patients were assessed in the PP analysis (small-bite group, n = 108; large-bite group, n = 108).

**Table 1:** Hernia rates until 5 years postop ESTOIH_ITT and PP analysis

Analysis	Short Stitch group	Long Stitch group	OR; 95% CI	p-value
1 yr ITT	3.3%, (7/210)	6.4%, (13/204)	1.95, 95% CI [0.77—5.05]	0.173
1 yr PP	4.2%, (7/165)	8.2%, (13/158)	2.02, 95% CI [0.79—5.21]	0.168
3yrs ITT	7.58%, (15/198)	10.45%, (21/201)	1.42, 95% CI [0.71—2.84]	0.316
3yrs PP	10.79%, (15/139)	15.67%, (21/134)	1.53, 95% CI [0.75—3.12]	0.233
5yrs ITT	9.14%, (16/175)	13.90%, (26/187)	1.60, 95% CI [0.82–3.10]	0.155
5yrs PP	14.81%, (16/108)	24.07%, (26/108)	1.82, 95% CI [0.91–3.63]	0.084

ITT: intention to treat anaylsis, PP: per protocol analysis, OR: odd ratio, CI: confidence intervall

From surgery until five years postoperatively, several subjects were not available for the five years examination in the short-stitch group due to the following reasons: 40 patients were lost to follow-up, 21 died, 12 withdrew, 18 patients received a relaparotomy and 31 cases prematurely completed the study due to other reasons compared to the long-stitch group: 23 lost to follow-up, 31 deaths, six withdrawals, 29 relaparotomies and 35 due to other reasons.

### Outcomes

#### Incisional hernias

The primary endpoint of the ESTOIH study, the incidence of hernias after one year postoperatively, as well as the 3-year incisional hernia results, have been published previously [19, 20], and the data are included in Table 1. In this report, we present the five-year findings.

**Fig. 2:**
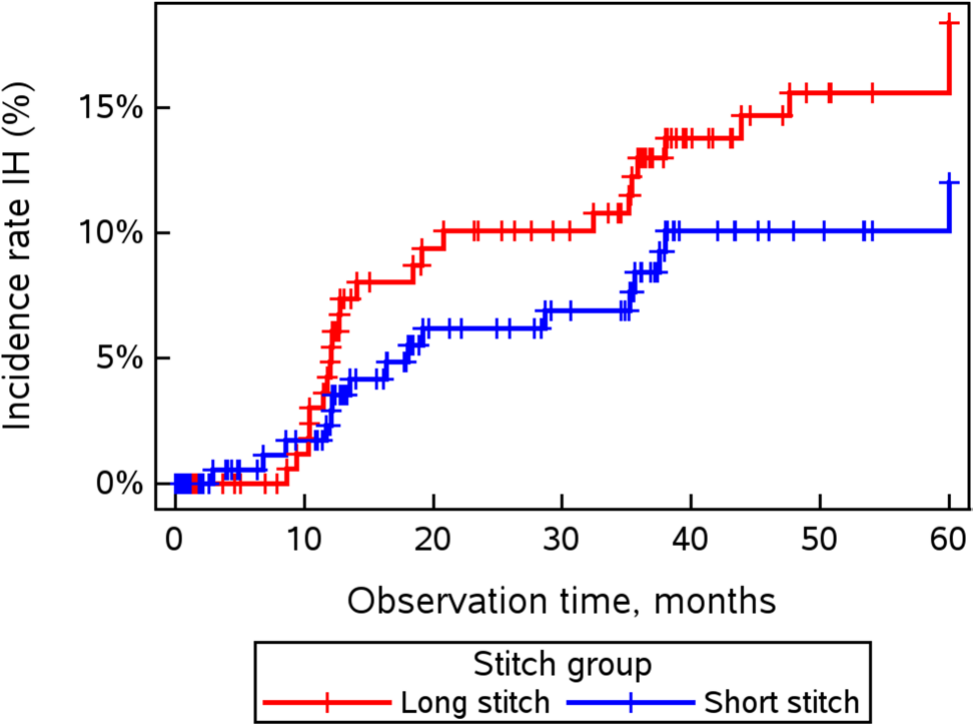
Incidence of IH until 5 years postop

The intention-to-treat analysis (ITT) indicated an incisional hernia rate of 9.14% (16/175 patients) for the short-stitch technique compared to 13.90% (26/187 patients) for the long-stitch technique (p = 0.155, OR 1.60; 95% CI [0.82–3.10]). In the per-protocol analysis (PP), the incisional hernia rate was 14.81% (16/108 patients) in the short-stitch group and 24.07% (26/108 patients) in the long-stitch group (p = 0.084, OR 1.82; 95% CI [0.91–3.63]), Table 1. As shown in Table 1, the incisional hernia rate in the short-stitch group was lower at every time point than that in the long-stitch group, but the difference was not statistically significant. Figure 2 shows the development of incisional hernias over time. A constant gap between the short-stitch and long-stitch techniques was observed from 1 to 5 years after surgery. A significant increase in the incisional hernia rate across both suture technique groups was seen in the ITT from 4.83% to 9.02% to 16.03% (p = 0.0183, p = 0.0063) and also in the per-protocol analysis from 6.19% to 13.18% to 19.44% (p = 0.0035, p = 0.0606) after 1 to 3 years and up to 5 years after surgery, respectively.

The majority of incisional hernias were diagnosed in the epigastric region (52%), followed by the umbilical (26%), subxiphoidal (7%), and infraumbilical regions (5%), with a comparable distribution in both stitch groups (Table 2). In total, 4 (10%) hernias were not categorised according to the EHS classification [21]. A similar distribution of hernia size was found in both stitch groups; most of the hernias were < 4 cm (48%), 13 (31%) were 4–10 cm, 2 were > 10 cm, and 7 were not reported (Table 2). Surgical treatment of incisional hernias was necessary more frequently in the short-stitch group (7/16, 43%) than in the long-stitch group (8/26, 30%) (Table 2).

**Table 2:** Characteristics of incisional hernias until 5 years FU

	Short Stitch group (n = 16)	Long stitch group (n = 26)	Total (n = 42)
**EHS Classification** **Localisation**			
Epigastric	9	13	22
Umbilical	4	7	11
Infraumbilical	1	1	2
Subxyphoidal	1	2	3
Suprapubic	0	0	0
Missing	1	3	4
**EHS Classification** **size (cm)**			
< 4 cm	8	12	20
≥ 4–10 cm	4	9	13
> 10 cm	1	1	2
Missing	3	4	7
**Need for surgical repair**			
Yes	7	8	15
No	9	18	27

### Quality of life

**Fig. 3:**
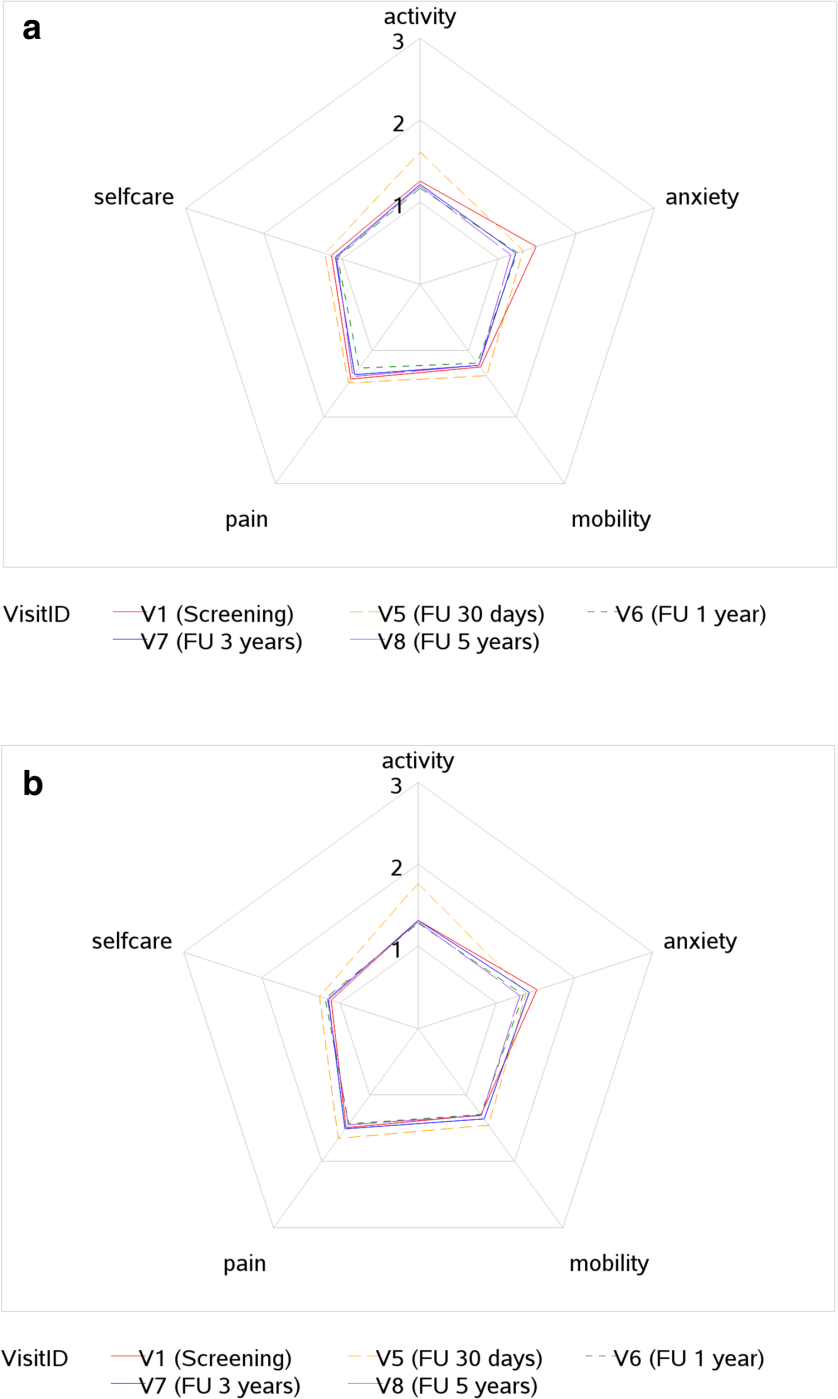
(**a**) EQ dimensions-short group until 5 years (**b**) EQ dimensions long group until 5 years

Table 3 shows the quality of life of the ESTOIH population from screening to five years postoperatively. The EQ-5D5L questionnaires [22] were completed by 167 of 216 patients (77%) at the five-year follow-up examination. Quality of life was higher in the short-stitch group than in the long-stitch group at each postoperative time point. The difference between the stitch groups regarding EQ-VAS values and the overall EQ Index was significant at 1 and 3 years after surgery (Tables 3 and 4). The quality of life was stable, remaining high from 1 to 5 years postoperatively within both treatment groups. Figures 3a and 3b show the different EQ dimensions, such as activity, pain, self-care, mobility, and anxiety, depending on time and stitch groups. At 30 days after surgery, pain was significantly lower in the short-stitch group than in the long-stitch group. Furthermore, the dimensions of pain and self-care were significantly better in the short-stitch group than in the long-stitch group at 1 year postoperatively, and the anxiety dimension was significantly worse in the long-stitch group at 3 years postoperatively. At five years after surgery, no differences were observed between the suture groups for any dimension.

**Table 3:** QoL EQ VAS Screening until 5 years postop

EQ scale (0–100)		N	Min	Q1	Median	Q3	Max	Mean	StdDev	p-Value
All	All	1192	0.00	65.00	80.00	90.00	100.00	74.82	18.38	
	Stitch group	617	0.00	65.00	80.00	90.00	100.00	76.23	18.24	
	Short stitch									
	Long stitch	575	10.00	60.00	75.00	90.00	100.00	73.32	18.42	
VisitID		367	0.00	65.00	80.00	90.00	100.00	75.26	18.12	
V1 (Screening)	All									
	Stitch group	187	0.00	65.00	80.00	90.00	100.00	75.90	18.19	
	Short stitch									0.4879
	Long stitch	180	10.00	60.00	80.00	90.00	100.00	74.59	18.08	
V5 (FU 30 days)	All	314	10.00	60.00	75.00	85.00	100.00	70.30	18.17	
	Stitch group	162	10.00	58.00	75.00	85.00	100.00	70.31	17.88	
	Short stitch									0.9900
	Long stitch	152	10.00	60.00	70.00	85.00	100.00	70.28	18.54	
V6 (FU 1 year)	All	288	10.00	70.00	80.00	90.00	100.00	77.59	17.37	
	Stitch group	152	10.00	70.00	81.00	92.50	100.00	80.44	16.70	
	Short stitch									**0.0031**
	Long stitch	136	20.00	65.00	80.00	90.00	100.00	74.41	17.61	
V7 (FU 3 years)	All	223	10.00	65.00	80.00	90.00	100.00	76.91	19.27	
	Stitch group	116	20.00	70.00	82.50	95.00	100.00	79.50	18.69	
	Short stitch									**0.0360**
	Long stitch	107	10.00	60.00	80.00	90.00	100.00	74.09	19.58	
	All	167	20.00	70.00	85.00	90.00	100.00	80.12	17.07	
V8 (FU 5 years)										
	Stitch group	86	20.00	75.00	85.00	95.00	100.00	81.81	15.56	0.1872
	Short stitch									
	Long stitch	87	20.00	70.00	80.00	90.00	100.00	78.32	18.47

**Table 4:** p-values of EQ index and different EQ dimensions until 5 years

**EQ Index**	Total	Mobility	Self-Care	Activity	Pain	Anxiety
Screening	0.4699	0.4376	0.7349	0.3843	0.4678	0.6495
30 days	0.1342	0.3643	0.4867	0.1436	**0.0438**	0.4199
1 year	**0.0411**	0.1317	**0.0279**	0.1294	**0.0376**	0.2227
3 year	**0.0346**	0.1477	0.3269	0.2352	0.1340	**0.0306**
5 year	0.3914	0.3749	0.1883	0.3098	0.6025	0.0998

## Discussion

This study represents the first randomised controlled trial (RCT) to report 5-year follow-up data comparing short-versus long-stitch techniques for midline abdominal wall closure. In total, 362 patients were included in the intention-to-treat (ITT) analysis, with 175 and 187 patients in the short-bite and long-bite groups, respectively. Our findings indicate that while the incisional hernia (IH) rate increased over time in both groups, the short-stitch group consistently exhibited a lower IH rate than the long-stitch group.

Importantly, both groups showed an increase in IH rates from 1 to 5 years postoperatively; however, the long-stitch group consistently exhibited higher IH rates at every time point. Kaplan–Meier analysis confirmed this observation, with a stable separation of the IH incidence curves between the two groups from 1 to 5 years, further suggesting that the short-stitch approach may offer a more durable protective effect against hernia formation.

The data presented here align with the growing body of evidence supporting the superior efficacy of the short-stitch technique, particularly when combined with an extra-long-term absorbable, elastic, monofilament poly-4-hydroxybutyrate (P4HB) suture. The very low IH rate observed in the short-stitch group at 5 years underscores the potential of this technique to achieve sustained results over time, even in long-term follow-up.

Furthermore, the patient-reported outcomes in this study also favoured the short-stitch group. The EQ-5D visual analogue scale (VAS) and EQ-5D index scores were significantly higher at 1 and 3 years in the short-stitch group, reflecting not only a reduced hernia burden but also an improvement in the overall quality of life. These findings reinforce the notion that surgical techniques that minimise postoperative complications, such as incisional hernia, can contribute to better long-term patient satisfaction and functional recovery.

It is important to distinguish between the effects of the suture technique and suture material. In the ESTOIH trial, both groups received the same extra-long-term absorbable elastic monofilament suture, allowing for a comparison of the technique alone. The lower hernia rate observed in the short-stitch group reflects the effect of the technique, while the low absolute hernia rates in both groups, relative to historical controls, may be partly attributed to the favourable material properties. When comparing the results of the ESTOIH study with those of other studies, our results seem particularly noteworthy. In the ESTOIH study, the 5-year IH rates in both arms were lower than the 1-year rates observed in the STITCH study [8]. This supports the hypothesis of a strictly standardised technique in combination with a highly elastic, ultralong resorbable suture material (Monomax®) used in the ESTOIH study compared to the suture material of polydioxanon (PDS®) used in the STITCH trial. Moreover, the hernia rate in the long-stitch group of the ESTOIH study was significantly lower than that reported in the suture-only group of the PRIMA trial (53.4%) after five years. In this study, a prophylactic mesh in the onlay and retrorectus positions was compared to a large bite closure using the all-in-one stitch technique with PDS®. Compared to the 1-year results of the ISAAC study, a historically controlled, single-arm, multicentre, prospective study evaluating the safety of MonoMax® with an IH rate of 14%, the 5-year data from the ESTOIH study are also remarkable. [14, 23, 24]. Taken together, the combination of a refined short-stitch technique and modern suture material may provide optimal outcomes for elective midline closure.

These comparisons highlight the progress in surgical techniques and material selection, with the short-stitch technique emerging as a more effective approach for reducing IH formation.

Suture materials likely play a relevant role in influencing these outcomes. The use of Monomax®, a slowly absorbable monofilament suture, has shown promising results in previous studies. Lai et al. reported less postoperative pain with the short-stitch technique than with the long-stitch technique using Monomax® [25]. These findings also reflect those of the ESTOIH study. Probst et al. demonstrated the suitability in recurrent hernia repairs, while Uske et al. and Sujatha et al. found lower IH rates when using Monomax® compared to non-resorbable polypropylene sutures [26–28]. These studies support the hypothesis that the combination of short-stitch techniques with advanced suture materials may contribute to lower complication rates and improved long-term outcomes.

In terms of economic impact, recent studies by Gokani et al.; Lwin et al. and Millbourn et al. have demonstrated the cost-effectiveness of the short-stitch technique [29–31]. The reduction in IH rates and the associated decrease in the need for reoperations can lead to significant long-term cost savings for both healthcare systems and patients. This potential economic advantage, coupled with the clinical benefits demonstrated in the present study, further strengthens the case for adopting the short-stitch technique as the preferred approach for abdominal wall closure.

Despite the available evidence, the short-stitch technique does not appear to be widely used internationally. Numerous surveys conducted in different countries have confirmed this situation. Barriers to implementation include ignorance of the scientific data, the argument of increased closure time, and failure to follow the recommendation to incorporate the short-bite technique in the updated EHS and AHS guidelines for abdominal wall closure in daily routine (32–36). In the survey conducted by Cochrun et al. (32), various problem areas of implementation were evident. These include the training and practice environment, as well as the associated decision-making of surgeons, which is primarily mediated by mentors and colleagues who either support or oppose the results, and the influence of peers, with anecdotal negative results often taking precedence over evidence-based results. In the UK national survey by Messenger et al. (33), the adoption of the short-stitch technique was also very low at 19.9%. The most common barriers to the use of small bites were a perceived lack of evidence and a perception of low personal incision hernia rates. Not surprisingly, a survey of colorectal surgeons in Spain (34) revealed that a closure technique using continuous sutures was employed in 96.23% of cases, with a single layer in 81.13% of cases, and a suture size of USP 1 in 58.49% of cases, suggesting the use of large-bite sutures only.

In the context of short-stitch method implementation, it can be concluded that only a combination of comprehensive education on the scientific evidence of the incisional hernia rate and closure methods, as well as on the technical aspects of the short-stitch closure procedure, in the context of workshops in the different surgical disciplines with open access surgery, can be achieved in the long term.

Several limitations of this study should be mentioned. First, despite the inherent challenges of long-term follow-up, 84% of the participants completed the 5-year assessment, with attrition rates balanced across the treatment arms, minimising the risk of systematic bias. Although follow-up rates were high and evenly distributed across groups, we acknowledge that some attrition may have been related to deteriorating health or reduced quality of life, an inherent limitation of long-term observational analyses.

Second, ensuring a uniform suture technique across trial centres and over time is inherently challenging. In the ESTOIH trial, the consistent application of the assigned techniques was supported by protocol-defined metrics, such as the suture length-to-wound length (SL:WL) ratio, complemented by structured training and regular site monitoring, underscoring the procedural integrity of this multicentre study.

Third, as reported in the initial publication, the trial was terminated after the interim analysis because of low recruitment and failure to achieve the predefined study aim. Consequently, the final sample size was below the target, limiting the statistical power of long-term comparisons. The absence of statistical significance at 5 years may reflect a smaller absolute difference and reduced power rather than a loss of effect.

Finally, in the 1-year multivariable analysis, a high BMI (≥ 30 kg/m^2^) emerged as a significant independent risk factor, whereas other comorbidities were not associated with hernia formation. Although comorbidities may change over time, the risk of incisional hernia is largely determined by perioperative factors. Therefore, longitudinal tracking of evolving comorbidities was not included in the ESTOIH trial protocol, and no conclusions can be drawn from our data regarding the role of evolving comorbidities during long-term follow-up.

In conclusion, the 5-year results of this study suggest that the short-stitch technique, particularly when combined with an extra-long-term absorbable, elastic, monofilament P4HB suture, offers significant advantages in terms of both clinical outcomes and patient-reported quality of life. While the difference in IH rates between the two techniques was not statistically significant at 5 years, the lower rates observed in the short-stitch group at every time point suggest a more favourable long-term outcome. These findings, along with supporting evidence from other studies, highlight the short-stitch technique as a promising approach for reducing incisional hernia formation and improving patient quality of life after abdominal wall closure following midline laparotomy.
